# Clinical outcomes of renin angiotensin system inhibitor-based dual antihypertensive regimens in chronic kidney disease: a network meta-analysis

**DOI:** 10.1038/s41598-023-32266-4

**Published:** 2023-04-07

**Authors:** Miseung Cho, Chang-Young Choi, Yeo Jin Choi, Sandy Jeong Rhie

**Affiliations:** 1 Graduate School of Converging Clinical & Public Health, Ewha Womans University, Seoul, 03760 Korea; 2 Department of Internal Medicine, Ajou University Medical Center, Suwon, 16499 Korea; 3 Department of Pharmacy, College of Pharmacy, Kyung Hee University, 26 Kyungheedae-Ro, Dongdamun-Gu, Seoul, 02447 Korea; 4 Department of Regulatory Science, Graduate School, Kyung Hee University, Seoul, 02447 Korea; 5 Institute of Regulatory Innovation through Science (IRIS), Kyung Hee University, Seoul, 02447, Korea; 6 College of Pharmacy, Graduate School of Pharmaceutical Sciences, Ewha Womans University, 52 Ewhayeodae-Gil, Seodamun-Gu, Seoul, 03760 Korea

**Keywords:** Therapeutics, Kidney diseases

## Abstract

This study comprehensively investigated clinical outcomes associated with renin angiotensin system inhibitor-based dual antihypertensive regimens in non-dialysis chronic kidney disease (CKD) patients**.** Keyword searches of databases were performed per PRISMA-NMA guidelines. Frequentist network meta-analysis were conducted with 16 head-to-head randomized controlled trials. The effect sizes of dichotomous and continuous variables were estimated with odds ratio (OR) and standard mean differences (SMD), respectively. The protocol is registered in PROSPERO (CRD42022365927). Dual antihypertensive regimens with combination of angiotensin receptor blockers (ARB) and calcium channel blockers (CCB) demonstrated substantially reduced odd of major cardiovascular disease (CVD) events over other regimens including angiotensin converting enzyme inhibitor (ACEI) monotherapy (OR 3.19) and ARB monotherapy (OR 2.64). Most significant reductions in systolic (SBP) and diastolic blood pressure (DBP) were observed with ARB-based CCB dual regimen over ACEI monotherapy (SMD 17.60 SBP and 9.40 for DBP), ACEI-based CCB regimen (SMD 12.90 for SBP and 9.90 for DBP), and ARB monotherapy (SMD 13.20 for SBP and 5.00 for DBP). However, insignificant differences were noticed for the odds of hyperkalemia, end stage renal disease progression, and all-cause mortality. ARB-based CCB regimen has the greatest benefits on BP reduction as well as major CVD risks in non-dialysis CKD patients.

## Introduction

Chronic kidney disease (CKD) is a progressive disorder characterized by renal insufficiency with an estimated glomerular filtration rate (eGFR) less than 60 mL/min/1.73 m^2^ for more than 3 months, responsible for 1.2 million mortalities^1–3^. Among numerous comorbidities, hypertension is one of the most common etiologic factors for CKD, and the prevalence increases with declining renal function, accounting for 60 to 90% of CKD patients^4^. Hypertension is considered as a dominant attributed comorbidity for end stage renal disease (ESRD), defined as eGFR < 15 mL/min/1.73 m^2^ or renal failure, as blood pressure (BP) increases with worsening renal insufficiency, which subsequently accelerates the disease progression^5^. Moreover, uncontrolled hypertension increases the risks of significant adverse cardiovascular outcomes such as myocardial infarction (MI), stroke, or sudden cardiac death, and CVD is designated for the leading cause of mortality in CKD patients indeed^6^. Thus, maintaining target BP goals in CKD patients is critical to improve clinical prognoses.

The updated 2021 Kidney Disease Improving Global Outcomes (KDIGO) guideline for the management of blood pressure in CKD recommends renin-angiotensin system inhibitors (RASi), either angiotensin converting enzyme inhibitors (ACEI) or angiotensin receptor blockers (ARB), as the first-line antihypertensive agents for non-dialysis CKD patients with elevated BP to inhibit markedly increased renin angiotensin aldosterone system (RAAS) activity induced by renal insufficiency^1,4,7^. According to the previous studies, both ACEI and ARB not only delayed CKD progression but also reduced CVD events including heart failure and cardiac death in patients with renal insufficiency^8^. However, most CKD patients require additional antihypertensive agents to RASi to maintain optimal BP as the disease progresses. According to a nationwide population study^9^, more than 50% of CKD patients were prescribed with at least 2 antihypertensive agents to manage hypertension. Although the current guidelines suggest dual antihypertensive treatment with RASi, either ACEIs or ARBs, in CKD patients with uncontrolled BP by the primary antihypertensive agent, the valid evidences on the optimal selection of add-on agent for RASi-based dual hypertension treatment regimens in CKD patients are currently limited^1,10^, and considering increased risk of adverse events (AE) secondary to substantial changes in pharmacokinetic and pharmacodynamic characteristics, there is an urgent need for the establishment of evidence-based guidance on optimal pharmacotherapy in patients with renal insufficiency^11^. Nevertheless, as CKD patients are considered as vulnerable patient populations, the clinical studies to support the selection of optimal RASi-based dual antihypertensive regimens in patients with renal insufficiency are currently lacking, consequently making clinical decision more perplex^12^. Therefore, this study aims to comprehensively assess clinical outcomes of diverse RASi-based dual antihypertensive regimens in non-dialysis CKD patients by conducting pair-wise comparisons utilizing Frequentist network meta-analysis methods to evaluate clinical benefits of each RASi-based dual antihypertensive regimen, thereby providing supporting evidences on the optimal RASi-based dual antihypertensive regimens in non-dialysis CKD patients to enhance prognosis.

## Results

### Study selection and characteristics

**Figure 1:**
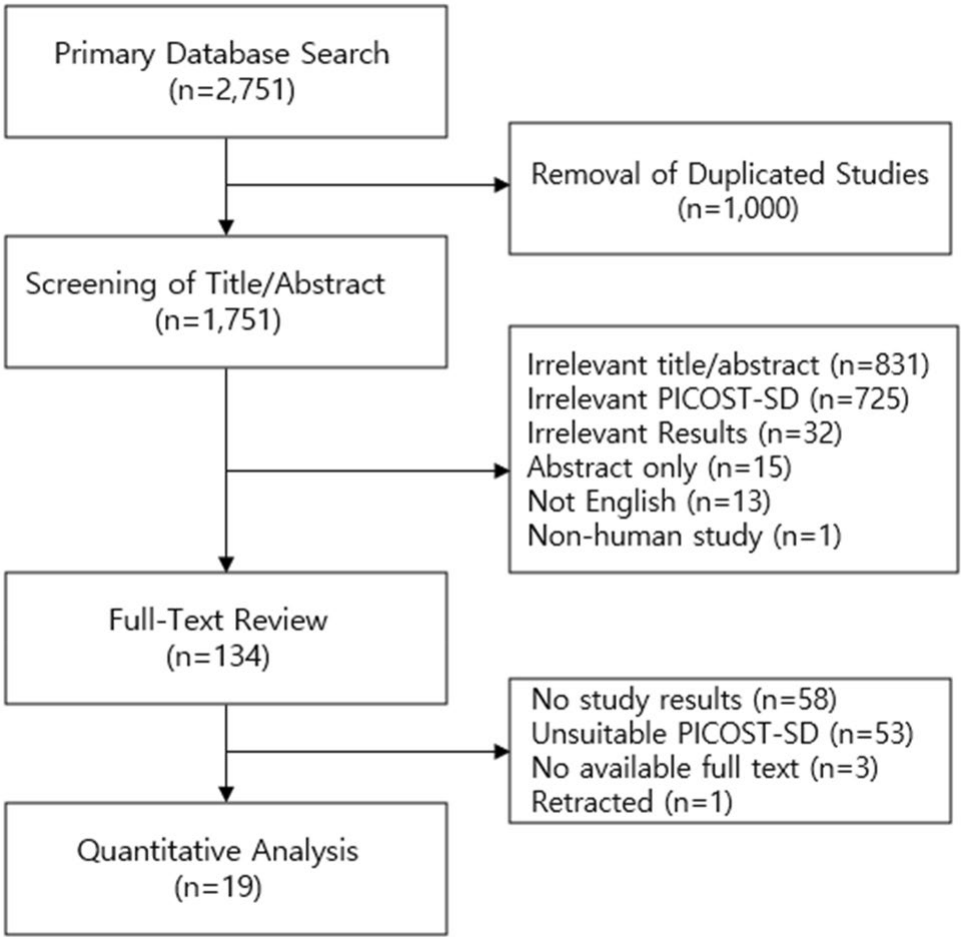
PRISMA plot.

**Figure 2:**
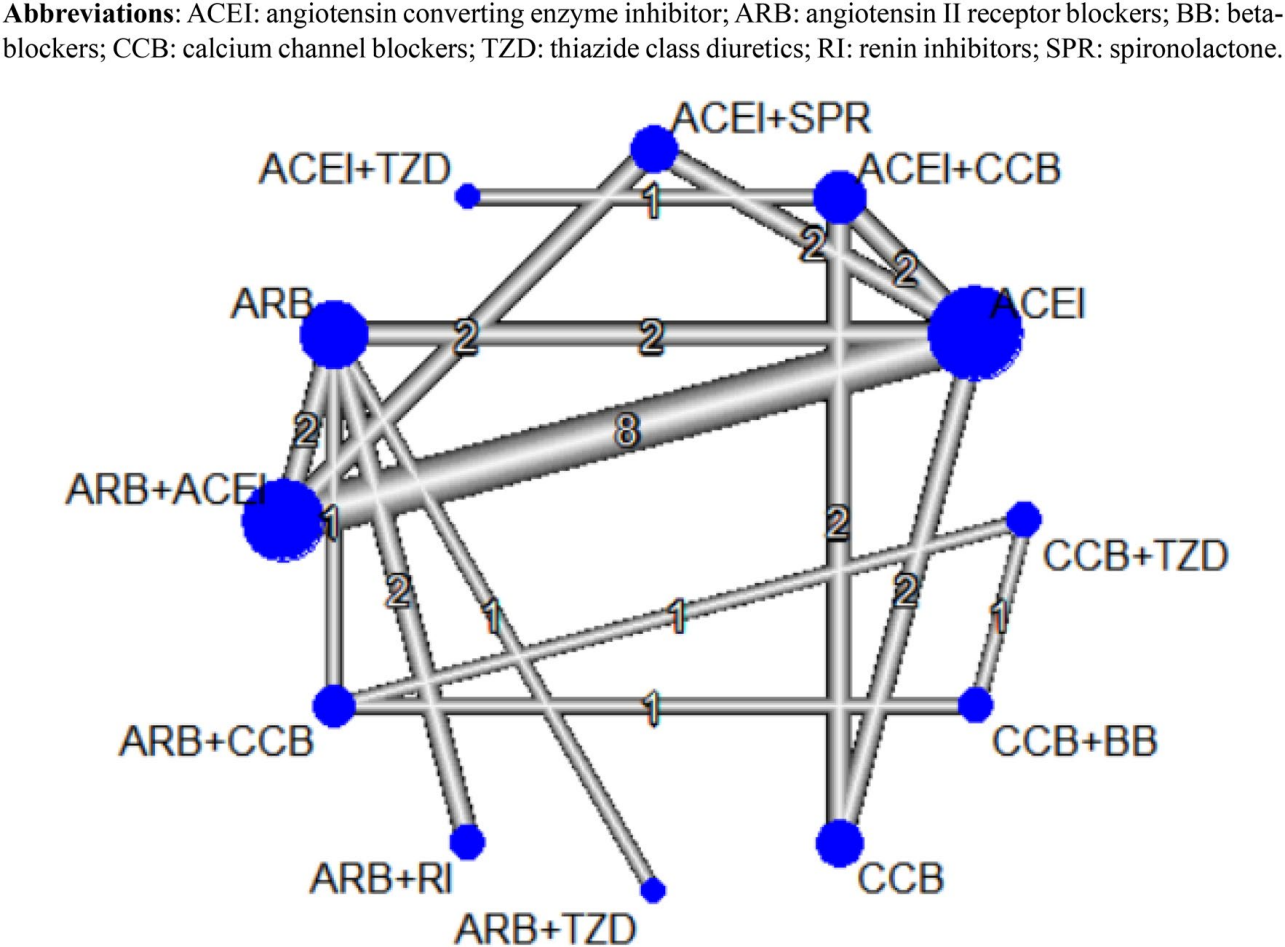
Network plot of included studies.

**Figure 3:**
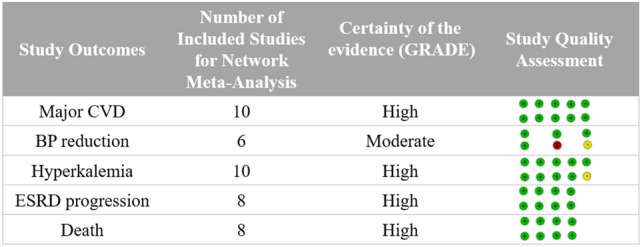
Quality Assessment of Included Studies and Outcomes.

The primary database search and study selection process per PRISMA and PRISMA-NMA guidelines are described in Fig. 1. The primary database search yielded 2.751 studies, and 134 studies were eligible for full-text review after exclusion of duplicates, irrelevant studies, abstracts, non-human study, and studies published in languages other than English. After the full-text review, a total of 16 head-to-head randomized controlled trials were included for quantitative network meta-analysis. The lists and characteristics of the eligible studies for network meta-analysis are summarized in Table 1. Twelve types of antihypertensive therapy regimens in 4,677 patients were included in the analysis: ACEI monotherapy^13–21^, ARB monotherapy^18,21–25^, CCB monotherapy^13,14^, ARB and ACEI combination^15–19,21^, ARB and CCB combination^24,26,27^, ARB and thiazide combination (TZD)^23,26^, ARB and renin inhibitor (RI) combination^22,25^, ACEI-based CCB combination^13,14,20,28^, ACEI and SPR combination^16,20^, ACEI and TZD combination^28^, CCB and beta-blocker combination (BB)^27^, and CCB and TZD combination^27^. The network plot of eligible studies is described in Fig. 2. The most studied head-to-head trial pair comparison was ARB and ACEI combination therapy versus ACEI monotherapy (8 studies). The results of study quality assessment are organized in Supplementary Figure S1. Majority of included studies (13 studies) had low risk of bias, suggesting high quality of the evidence. The quality of generated evidences of each outcome based on GRADE approach is described in Fig. 3. The possibility of publication bias may present only in hyperkalemia risk based on the results of Egger’s test (*P* values < 0.05). All study outcomes had I^2^ index < 50% and *P* values > 0.05 for Cochran Q statistics, implying low risk of heterogeneity and inconsistency.

**Table 1:** Study characteristics.

References	Treatment (Drug Class)		Patients	DM (Y/N)	Elderly (Y/N)	Outcomes	F/U period
Herlitz et al.^13^	Ramipril + Felodipine	(ACEI + CCB)	51	N	–	SBP, DBP, ESRD, major CVD, death	2 years
	Ramipril	(ACEI)	53				
	Felodipine	(CCB)	54				
MacGregor et al.^14^	Quinapril + Amlodipine	ACEI + CCB	17	N	–	ESRD, hyperkalemia, major CVD, death	4 years
	Quinapril	ACEI	28				
	Amlodipine	CCB	28				
Kanno et al.^15^	Candesartan + ACEI	(ARB + ACEI)	45	N	–	SBP, DBP, ESRD	3 years
	ACEI	(ACEI)	45				
Fogari et al.^26^	Candesartan + Manidipine	(ARB + CCB)	87	Y	–	SBP, DBP	24 weeks
	Candesartan + HCTZ	(ARB + TZD)	87				
Parving et al.^22^	Losartan + Aliskiren	(ARB + RI)	301	Y	–	hyperkalemia, major CVD	24 weeks
	Losartan	(ARB)	298				
Abe et al.^23^	Losartan + HCTZ	(ARB + TZD)	30	–	–	SBP, DBP	24 weeks
	Losartan	(ARB)	30				
Mehdi et al.^16^	Spironolactone + ACEI	(ACEI + SPR)	27	Y	–	major CVD	48 weeks
	Losartan + ACEI	(ARB + ACEI)	26				
	ACEI	(ACEI)	27				
Bakris et al.^28^	Benazepril + Amlodipine	(ACEI + CCB)	335	–	Y	ESRD, hyperkalemia, major CVD, Death	3 years
	Benazepril + HCTZ	(ACEI + TZD)	309				
Imai et al.^17^	Olmesartan + ACEI	(ARB + ACEI)	205	Y	–	ESRD, hyperkalemia, major CVD, Death	3 years
	ACEI	(ACEI)	209				
Fernandez Juarez et al.^18^	Irbesartan + Lisinopril	(ARB + ACEI)	70	Y	–	SBP, DBP, ESRD, hyperkalemia	4 years
	Irbesartan	(ARB)	28				
	Lisinopril	(ACEI)	35				
Rakugi et al.^27^	ARB + Benidipine	(ARB + CCB)	287	–	–	Major CVD, hyperkalemia, death	3 years
	BB + Benidipine	(CCB + BB)	283				
	Benidipine + Thiazide	(CCB + TZD)	264				
Torres et al.^19^	Telmisartan + Lisinopril	(ARB + ACEI)	244	N	–	SBP, DBP, ESRD, hyperkalemia, death	5 years
	Lisinopril	(ACEI)	242				
Van Buren et al.^20^	Losartan + Lisinopril	(ARB + ACEI)	26	Y	–	hyperkalemia	48 weeks
	Spironolactone + Lisinopril	(ACEI + SPR)	27				
	Lisinopril	(ACEI)	27				
Kim-Mitsuyama et al.^24^	Olmesartan + Amlodipine or Azelnidipine	(ARB + CCB)	172	–	Y	ESRD, hyperkalemia, major CVD, Death	3 years
	Olmesartan	(ARB)	181				
Soji et al.^25^	ARB + Aliskiren	(ARB + RI)	42	–	–	ESRD, major CVD	1 year
	ARB	(ARB)	41				
Saglimbene et al.^21^	ARB + ACEI	(ARB + ACEI)	416	Y	–	ESRD, hyperkalemia, major CVD, Death	4 years

ACEI, angiotensin converting enzyme inhibitor; ARB, angiotensin receptor blocker; BB, beta-blocker; CCB, calcium channel blocker; CVD, cardiovascular disease; DBP, diastolic blood pressure; ESRD, end stage renal disease; F/U, follow up; RI, renin inhibitor; SBP, systolic blood pressure; SPR, spironolactone; TZD, thiazide.

### Study outcomes from network meta-analysis

**Figure 4:**
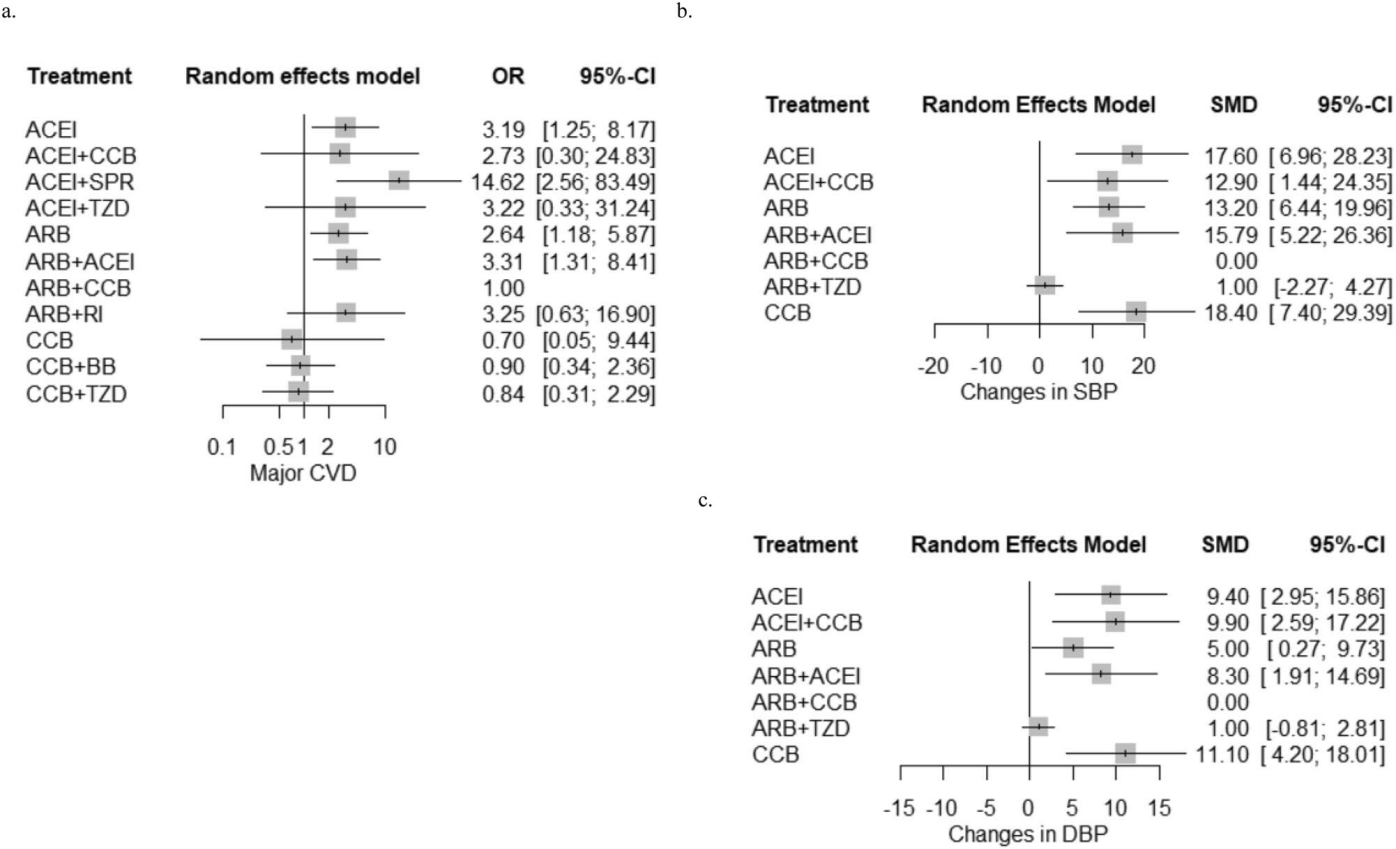
Cardiovascular outcomes of antihypertensive interventions in CKD patients. (**a**) Major CVD events, (**b**) changes in SBP, and (**c**) changes in DBP.

**Figure 5:**
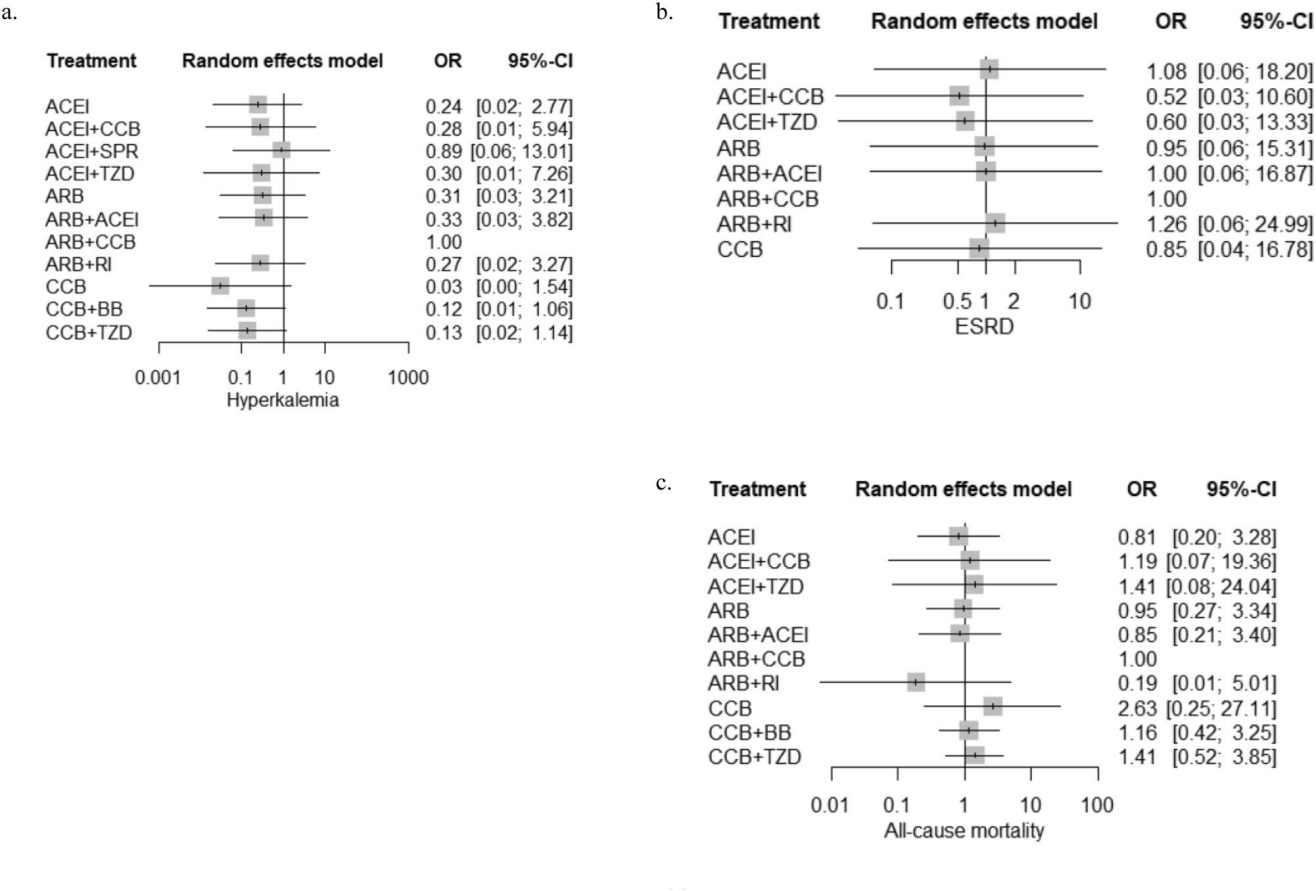
Other clinical outcomes of antihypertensive interventions in CKD patients. (**a**) Hyperkalemia, (**b**) ESRD progression, and (**c**) all-cause mortality.

The odd of major CVD events was significantly higher in ACEI monotherapy (OR 3.19; 95% CI 1.25–8.17, *p* = 0.0156), ACEI and SPR combination (OR 14.62; 95% CI 2.56- 83.49*, P* = 0.0026), ARB monotherapy (OR 2.64; 95% CI 1.18–5.87 *P* = 0.0177), and ARB and ACEI combination (OR 3.31; 95% CI 1.31–8.41, *P* = 0.0117) than ARB-based CCB combination therapy (Fig. 4a and Table 2). Similar odds of major CVD event were noticed in other RASi-based dual antihypertensive regimens including ACEI-based TZD combination and ACEI-based CCB combination when compared to ARB-based CCB combination. However, ACEI-based SPR combination therapy demonstrated markedly higher odds of major CVD events than ARB monotherapy, ARB-based CCB combination regimen, CCB monotherapy, CCB and BB combination and CCB and TZD combination (*P* < 0.05) (Table 2). ARB-based CCB combination therapy demonstrated substantially greater reduction in SBP than ACEI monotherapy (SMD 17.60; 95% CI 6.96–28.23; *P* = 0.0035), ACEI and CCB combination (SMD 12.90; 95% CI 1.44–24.35; *P* = 0.0499), ARB monotherapy (SMD 13.20; 95% CI 6.44–19.96; *P* = 0.0012), ARB and ACEI combination (SMD 15.79; 95% CI 5.22–26.36; *P* = 0.0086) and CCB monotherapy (SMD 18.40; 95% CI 7.40–29.39; *P* = 0.0039) (Fig. 4b and Table 3). Significantly lowered DBP was also observed in ARB-based CCB combination regimen when compared to ACEI monotherapy (SMD 9.40; 95% CI 2.95- 15.86; *P* = 0.0043), ACEI-based CCB combination regimen (SMD 9.90; 95% CI 2.59–17.22, *P* = 0.0080), ARB monotherapy (SMD 5.00; 95% CI 0.27–9.73; *P* = 0.0382), ARB and ACEI combination (SMD 8.30; 95% CI 1.91–14.69; *P* = 0.109) and CCB monotherapy (SMD 11.10; 95% CI 4.20–18.01; *P* = 0.0016) (Fig. 4c and Table 4). The BP control capacity was similar between ARB-based CCB combination and ARB-based TZD combination. The odd of hyperkalemia is statistically insignificant among diverse RASi-based antihypertensive treatment when referenced with ARB-based CCB combination therapy (Fig. 5a and Table 5). However, the indirect comparison analysis revealed substantially higher odd of hyperkalemia with ACEI-based SPR combination regimens than CCB monotherapy (*p* < 0.05) (Table 5). The odds of ESRD diagnosis and all-cause mortality were insignificant among antihypertensive treatment regimens (Fig. 5b,c and Tables 6, 7). The SUCRA analysis on ranking of antihypertensive treatment regimens for each outcome is summarized in Table 8. ARB-based CCB combination regimen demonstrated relatively better prognosis for major CVD events and BP controls among antihypertensive treatment regimens.

**Table 2:** Net league table of major CVD events.

ACEI	1.07 [0.11; 9.97]	0.13 [0.02; 1.21]		1.04 [0.61; 1.77]	0.96 [0.67; 1.37]			5.38 [0.25; 117.25]		
1.17 [0.16; 8.60]	ACEI + CCB		0.85 [0.49; 1.46]					3.24 [0.13; 81.31]		
0.22 [0.05; 0.96]	0.19 [0.02; 2.27]	ACEI + SPR			3.43 [0.62; 18.84]					
0.99 [0.13; 7.84]	0.85 [0.49; 1.46]	4.54 [0.35; 58.37]	ACEI + TZD							
1.20 [0.74; 1.96]	1.04 [0.13; 8.10]	**5.54** **[1.18; 26.05]**	1.22 [0.15; 10.24]	ARB	0.71 [0.43; 1.17]	**2.64** **[1.18; 5.87]**	0.81 [0.19; 3.42]			
0.96 [0.67; 1.37]	0.82 [0.11; 6.26]	4.41 [0.99; 19.47]	0.97 [0.12; 7.93]	0.80 [0.49; 1.28]	ARB + ACEI					
**3.19** **[1.25; 8.17]**	2.73 [0.30; 24.83]	**14.62** **[2.56; 83.49]**	3.22 [0.33; 31.24]	**2.64** **[1.18; 5.87]**	**3.31** **[1.31; 8.41]**	ARB + CCB			1.11 [0.42; 2.93]	1.19 [0.44; 3.24]
0.98 [0.21; 4.49]	0.84 [0.07; 10.34]	4.50 [0.54; 37.19]	1.00 [0.08; 12.90]	0.81 [0.19; 3.42]	1.02 [0.22; 4.64]	0.31 [0.06; 1.60]	ARB + RI			
4.54 [0.40; 51.27]	3.89 [0.33; 45.54]	**20.83** **[1.21; 359.73]**	4.59 [0.37; 56.93]	3.76 [0.32; 44.53]	4.72 [0.41; 54.67]	1.43 [0.11; 19.17]	4.64 [0.27; 81.05]	CCB		
3.55 [0.91; 13.67]	3.04 [0.27; 33.83]	**16.27** **[2.22; 119.33]**	3.58 [0.30; 42.34]	2.93 [0.84; 10.30]	3.69 [0.97; 14.12]	1.11 [0.42; 2.93]	3.62 [0.54; 24.46]	0.78 [0.05; 12.50]	CCB + BB	1.07 [0.38; 2.99]
3.79 [0.96; 14.98]	3.25 [0.29; 36.65]	**17.40** **[2.33; 129.87]**	3.83 [0.32; 45.86]	3.94 [0.87; 11.30]	**3.94** **[1.00; 15.48**]	1.19 [0.44; 3.24]	3.87 [0.56; 26.60]	0.83 [0.05; 13.52]	1.07 [0.38; 2.99]	CCB + TZD

ACEI, angiotensin converting enzyme inhibitors; ARB, angiotensin receptor blockers; BB, beta-blocker; CCB, calcium channel blockers; CVD, cardiovascular disease; RI, renin inhibitor; SPR, spironolactone; TZD, thiazide diuretics.

Statistically significant values are expressed in bold.

**Table 3:** Net league table of changes in SBP.

ACEI	4.70 [− 0.49; 9.89]	2.00 [− 7.83; 11.93]	1.91 [− 1.24; 5.06]			
4.70 [− 0.49; 9.89]	ACEI + CCB					− 0.800 [− 4.88; 3.28]
4.40 [− 4.31; 13.10]	− 0.30 [− 10.44; 9.83]	ARB	− 4.00 [− 13.05; 5.05]		12.20 [5.57; 18.83]	**− 5.50** **[− 10.46; − 0.54]**
1.91 [− 1.24; 5.06]	− 2.79 [− 8.86; 3.28]	− 2.49 [− 11.06; 6.09]	ARB + ACEI			
**17.60** **[5.80; 29.40]**	**12.90** **[0.00; 25.79]**	**13.20** **[5.24; 21.16]**	**15.69** **[3.98; 27.39]**	ARB + CCB	− 1.00 [− 5.42; 3.42]	
**16.60** **[5.65; 27.54]**	**11.90** **[− 0.21; 24.00]**	**12.20** **[5.57; 18.83]**	**14.69** **[3.85; 25.52**	− 1.00 [− 5.42; 3.42]	ARB + TZD	
− 0.80 [− 4.88; 3.28]	**− 5.50** **[− 10.46; − 0.54]**	− 5.20 [− 14.81; 4.42]	− 2.71 [− 7.87; 2.45]	**− 18.40** **[− 30.88; − 5.91]**	**− 17.40** **[− 29.07; − 5.72]**	CCB

ACEI, angiotensin converting enzyme inhibitors; ARB, angiotensin receptor blockers; CCB, calcium channel blockers; SBP, systolic blood pressure; SMD, standard mean difference; TZD, thiazide diuretics.

Statistically significant values are expressed in bold.

**Table 4:** Net league tables of changes in DBP.

ACEI	− 0.50 [− 3.94; 2.94]	3.00 [− 2.13; 8.13]	1.10 [− 0.51; 2.71]			− 1.70 [− 4.16; 0.76]
− 0.50 [− 3.94; 2.94]	ACEI + CCB					− 1.20 [− 3.78; 1.38]
**4.41** **[0.01; 8.80]**	4.91 [− 0.68; 10.49]	ARB	− 4.00 [− 8.49; 0.49]		4.00 [− 0.37; 8.37]	
1.10 [− 0.51; 2.71]	1.60 [− 2.20; 5.40]	− 3.30 [− 7.60; 0.99]	ARB + ACEI			
**9.41** **[2.95; 15.86]**	**9.91** **[2.59; 17.22]**	**5.00** **[0.27; 9.73]**	**8.30** **[1.91; 14.69]**	ARB + CCB	− 1.00 [− 2.81; 0.81]]	
**8.41** **[2.21; 14.60]**	**8.91** **[1.81; 16.00]**	4.00 [− 0.37; 8.37]	**7.30** **[1.17; 13.43]**	− 1.00 [− 2.81; 0.81]	ARB + TZD	
− 1.70 [− 4.16; 0.76]	− 1.20 [− 3.78; 1.38]	**− 6.11** **[− 11.14;− 1.07]**	− 2.80 [− 5.74; 0.13]	**− 11.11** **[− 18.01; − 4.20]**	**− 10.11** **[− 16.77; − 3.44]**	CCB

ACEI, angiotensin converting enzyme inhibitors; ARB, angiotensin receptor blockers; CCB, calcium channel blockers; DBP, diastolic blood pressure; SMD, standard mean difference; TZD, thiazide diuretics.

Statistically significant values are expressed in bold.

**Table 5:** Net league table of hyperkalemia.

ACEI	0.85 [0.14; 5.22	**0.07** **[0.01; 0.41]**		0.88 [0.39; 2.04]	0.72 [0.47; 1.10]			7.82 [0.37; 165.84]		
0.85 [0.14; 5.22]	ACEI + CCB		0.95 [0.36; 2.47]					9.19 [0.40; 212.51]		
**0.27** **[0.08; 0.87]**	0.32 [0.04; 2.74]	ACEI + SPR			1.72 [0.52; 5.76]					
0.81 [0.10; 6.27]	0.95 [0.36 2.47]	2.99 [0.28; 41.67]	ACEI + TZD							
0.77 [0.37; 1.62]	0.90 [0.13; 6.42]	2.85 [0.76; 10.72]	0.95 [011; 8.45]	ARB	1.03 [0.48; 2.22]	0.31 [0.03; 3.22]	1.15 [0.48; 2.77]			
0.72 [0.47; 1.10]	0.85 [0.13; 5.47]	2.68 [0.87; 8.28]	0.90 [0.11; 7.27]	0.94 [0.46; 1.93]	ARB + ACEI					
0.24 [0.02; 2.77]	0.28 [0.01; 5.94]	0.89 [0.06; 13.01]	0.30 [0.01; 7.26]	0.31 [0.03; 3.22]	0.33 [0.03; 3.82]	ARB + CCB			8.09 [0.94; 69.21]	7.54 [0.88; 64.56]
0.89 [0.28; 2.80]	1.04 [0.12; 8.93]	3.29 [0.67; 16.11]	1.10 [0.01; 11.55]	1.15 [0.48; 2.77]	1.23 [0.40; 3.82]	3.69 [0.31; 44.42]	ARB + RI			
7.82 [0.37; 165.84]	9.19 [0.40; 212.51]	**29.02** **[1.10; 763.07]**	9.71 [0.36; 258.71]	10.18 [0.44; 235.83]	510.84 [0.49; 236.51]	32.52 [0.65; 1626.11]	8.82 [0.34; 230.54]	CCB		
1.95 [0.08; 50.36]	2.29 [0.06; 94.86]	7.22 [0.23; 223.64]	2.41 [0.05; 113.02]	2.53 [0.11; 60.13]	2.69 [0.10; 69.39]	8.09 [0.94; 69.217]	2.19 [0.08; 58.72]	0.25 [0.00; 21.56]	CCB + BB	0.93 [0.55; 15.70]
1.81 [0.07; 46.98]	2.13 [0.06; 88.48]	6.73 [0.22; 208.60]	2.25 [0.05; 105.42]	2.36 [0.10; 56.08]	2.51 [0.10; 64.72]	7.54 [0.88; 64.56]	2.05 [0.08; 54.78]	0.23 [0.00; 20.11]	0.93 [0.06; 15.70]	CCB + TZD

ACEI, angiotensin converting enzyme inhibitors; ARB, angiotensin receptor blockers; BB, beta-blocker; CCB, calcium channel blockers; RI, renin inhibitor; SPR, spironolactone; TZD, thiazide diuretics.

Statistically significant values are expressed in bold.

**Table 6:** Net league table of ESRD progression.

ACEI	2.12 [0.73; 6.20]		1.08 [0.61; 1.91]	1.08 [0.85; 1.36]			1.27 [0.49; 3.27]
2.09 [0.72; 6.10]	ACEI + CCB	0.86 [0.43; 1.74]					0.61 [0.22; 1.70]
1.80 [0.50; 6.48]	0.86 [0.43; 1.74]	ACEI + TZD					
1.14 [0.68; 1.89]	0.54 [0.17; 1.78]	0.63 [0.16; 2.50]	ARB	0.91 [0.52; 1.58]	0.95 [0.06; 15.31]	0.75 [0.25; 2.26]	
1.08 [0.85; 1.36]	0.52 [0.17; 1.54]	0.60 [0.16; 2.20]	0.95 [0.57; 1.57]	ARB + ACEI			
1.08 [0.06; 18.20]	0.52 [0.03; 10.60]	0.60 [0.03; 13.33]	0.95 [0.06; 15.31]	1.00 [0.06; 16.87]	ARB + CCB		
0.86 [0.26; 2.87]	0.41 [0.08; 2.06]	0.48 [0.08; 2.77]	0.75 [0.25; 2.26]	0.79 [0.24; 2.66]	0.79 [0.04; 15.78]	ARB + RI	
1.13 [0.49; 3.27]	0.61 [0.22; 1.67]	0.70 [0.20; 2.42]	1.12 [0.38; 3.28]	1.17 [0.44; 3.12]	1.17 [0.06; 23.15]	1.48 [0.32; 6.88]	CCB

ACEI, angiotensin converting enzyme inhibitors; ARB, angiotensin receptor blockers; CCB, calcium channel blockers; ESRD, end stage renal disease; RI, renin inhibitor; TZD, thiazide diuretics.

**Table 7:** Net league table of all-cause mortality.

ACEI	0.59 [0.03; 10.14]		0.79 [0.41; 1.52]	0.96 [0.61; 1.52]			0.32 [0.05; 2.09]		
0.68 [0.06; 7.56]	ACEI + CCB	0.85 [0.49; 1.46]					0.45 [0.07; 3.02]		
0.58 [0.05; 6.81]	0.85 [0.49; 1.46]	ACEI + TZD							
0.86 [0.47; 1.57]	1.26 [0.11; 15.12]	1.49 [0.12; 18.88]	ARB	1.03 [0.55; 1.92]	0.95 [0.27; 3.34]	5.08 [0.24; 106.35]			
0.95 [0.60; 1.51]	1.41 [0.12; 16.35]	1.66 [0.13; 20.44]	1.12 [0.62; 2.00]	ARB + ACEI					
0.81 [0.20; 3.28]	1.19 [0.07; 19.36]	1.41 [0.08; 24.04]	0.95 [0.27; 3.34]	0.85 [0.21; 3.40]	ARB + CCB			0.86 [0.31; 2.40]	0.71 [0.26; 1.93]
4.35 [0.20; 96.55]	6.40 [0.13; 324.96]	7.55 [0.14; 397.53]	5.08 [0.24; 106.35]	4.55 [0.21; 100.72]	5.36 [0.20; 143.89]	ARB + RI			
0.31 [0.05; 2.01]	0.46 [0.07; 2.99]	0.54 [0.08; 3.81]	0.36 [0.05; 2.59]	0.32 [0.05; 2.22]	0.38 [0.04; 3.93]	0.07 [0.00; 2.66]	CCB		
0.70 [0.12; 3.95]	1.03 [0.06; 19.99]	1.21 [0.06; 24.75]	0.82 [0.16; 4.14]	0.73 [0.13; 4.10]	0.86 [0.31; 2.40]	0.16 [0.01; 5.04]	2.26 [0.18; 28.93]	CCB + BB	0.82 [031; 2.17]
0.57 [0.10; 3.20]	0.85 [0.05; 16.33]	1.00 [0.05; 20.22]	0.67 [0.13; 3.36]	0.60 [0.11; 3.33]	0.71 [0.26; 1.93]	0.13 [0.00; 4.12]	1.86 [0.15; 23.60]	0.82 [0.31; 2.17]	CCB + TZD

ACEI, angiotensin converting enzyme inhibitors; ARB, angiotensin receptor blockers; BB, beta-blocker; CCB, calcium channel blockers; RI, renin inhibitor; TZD, thiazide diuretics.

**Table 8:** Surface under the cumulative ranking curve (SUCRA) and treatment ranking.

SBP		DBP					
Antihypertensive Regimens	SUCRA	Antihypertensive Regimens	SUCRA				
**ARB + CCB**	**9.396**	**ARB + CCB**	**0.9716**				
ARB + TZD	0.8824	ARB + TZD	0.8468				
ARB	0.5565	ARB	0.6522				
ACEI + CCB	0.4810	ARB + ACEI	0.4594				
ARB + ACEI	0.3681	ACEI	0.2754				
ACEI	0.1615	ACEI + CCB	0.2441				
CCB	0.1109	CCB	0.0516				

Major CVD		Hyperkalemia		ESRD		Death	
Antihypertensive Regimens	SUCRA	Antihypertensive Regimens	SUCRA	Antihypertensive Regimens	SUCRA	Antihypertensive Regimens	SUCRA
CCB + TZD	0.8135	CCB	0.8909	ACEI + CCB	0.8082	ARB + RI	0.8515
CCB	0.7938	CCB + BB	0.6752	ACEI + TZD	0.6876	ACEI	0.6268
CCB + BB	0.7924	CCB + TZD	0.6604	CCB	0.5115	ARB + ACEI	0.5910
**ARB + CCB**	**0.7730**	ACEI	0.6080	ARB	0.4724	**ARB + CCB**	**0.5242**
ARB	0.4530	ARB + RI	0.5256	**ARB + CCB**	**0.4616**	ARB	0.5180
ACEI + CCB	0.4485	ACEI + CCB	0.4973	ARB + ACEI	0.4304	ACEI + CCB	0.4867
ACEI + TZD	0.3664	ACEI + TZD	0.4744	ACEI	0.3201	CCB + BB	0.4537
ARB + RI	0.3653	ARB	0.4559	ARB + RI	0.3082	ACEI + TZD	0.3981
ACEI	0.3418	ARB + ACEI	0.4146			CCB + TZD	0.3578
ARB + ACEI	0.3135	**ARB + CCB**	**0.1637**			CCB	0.1923
ACEI + SPR	0.0387	ACEI + SPR	0.1341				

Bolded antihypertensive treatment regimen indicates the reference of the network meta-analysis. ACEI, angiotensin converting enzyme inhibitor; ARB, angiotensin receptor blockers; BB, beta-blockers; CCB, calcium channel blockers; CKD, chronic kidney disease; CVD, cardiovascular disease; DBP, diastolic blood pressure; DM, diabetes mellitus; ESRD, end-stage renal disease; RI, renin inhibitor; SBP, systolic blood pressure; TZD, thiazide diuretics; SPR, spironolactone.

## Discussion

Hypertension is the most prevalent comorbidity etiologic for CKD diagnosis and progression^4^. Uncontrolled hypertension not only increases the risk of major CVD events such as MI, stroke and cardiac death but also predisposes patients to the elevated risk of ESRD diagnosis, subsequently elevating the medication burdens associated with ESRD complications such as anemia, mineral bone disorder, electrolyte imbalance and uremia^29^. Nonetheless, implementation of optimal antihypertensive pharmacotherapy in CKD patients is still challenging due to limited number of evidences. Hence, this study evaluated the clinical outcomes associated with diverse RASi-based antihypertensive regimens in non-dialysis CKD patients to establish evidences on the optimal antihypertensive pharmacotherapy.

According to a previous network meta-analysis evaluating clinical outcomes of diverse antihypertensive monotherapy regimens, ACEI monotherapy has superior benefits on prevention of kidney disease and cardiovascular death over other regimens including ARB monotherapy, BB monotherapy and CCB monotherapy in non-dialysis CKD patients^30^. In this study, however, the risk of major CVD events was considerably elevated with RASi monotherapy, implying that either ACEI or ARB monotherapy may not be sufficient to control BP and major CVD risk in many CKD patients. The previous studies evaluating prescribing patterns of antihypertensive agents indeed revealed that more than 70% of CKD patients were prescribed at least 2 antihypertensives^9,31^. The types of dual antihypertensive regimens, however, were variable in these studies due to the obscurity on the optimal antihypertensive combination regimens in non-dialysis CKD patients^9,31^. Based on the treatment ranking results from SUCRA, ARB-based CCB combination had the lowest risk of major CVD events, followed by ACEI-based CCB combination and ACEI-based TZD combination (Table 3), and this may be correlated with the greatest BP reduction effects. Although this study demonstrated comparable reduction of major CVD risk and BP between RASi-based TZD combination and ARB-based CCB combination, the evidences suggest superior benefits of RASi-based CCB on prevention of cardiovascular death over RASi-based TZD in hypertensive patients with high risk for major CVD events, indicating potential advantage of ARB-based CCB combination regimen in non-dialysis CKD patients^32^. Moreover, considering that the diuretic efficacy of TZD worsens with decreased renal function^33^, RASi-based TZD combination may not be a promising choice for in patients with advanced CKD.

One of the concerns associated with RASi treatment is elevated risk of hyperkalemia^1,10^. Hyperkalemia induces cardiac arrhythmia, which consequently increases the risks of hospitalization and sudden cardiac death^10^. Both JNC8 and KDIGO guidelines restrict any combination of ACEI, ARB and direct RI in non-dialysis CKD patients based on the strong evidences of increased harm from hyperkalemia and acute kidney injury over cardiovascular and renal benefits^1,10^. Nonetheless, no significant difference in the risk of hyperkalemia was noticed among RASi-based antihypertensive treatment regimens, except RASi-based SPR combination treatment, when compared to the combination regimen of ARB with CCB. Interestingly, similar hyperkalemia risk was observed in concomitant administration of ACEI and ARB combination (OR 0.38; 95% CI 0.03–4.94) and non-RASi-based antihypertensive treatment including CCB monotherapy (OR 0.06; 95% CI 0.00–2.26), CCB and BB combination (OR 0.84; 95% CI 0.22–3.17) and CCB and TZD combination (OR 1.28; 95% CI 0.37- 4.42) in this study. Although the evident mechanism for similar hyperkalemia risks regardless of the diverse combination of antihypertensive agents is yet to be determined, disease-specific factor may have superior influences on hyperkalemia risk over RASi use in CKD patients^34^. Renal insufficiency itself is a compelling risk factor of hyperkalemia as the hyperkalemia incidence and severity increase with CKD progression, and a previous study revealed that the degree of renal insufficiency manifested by elevated serum creatinine level was the strongest positive correlation factor with serum potassium over other contributing factors such as diabetes, RASi use, and age^35^. However, the scarcity of evidences in CKD patients may hinder investigation of evident hyperkalemia risk from pharmacotherapy notwithstanding renal function. Hence, further studies investigating hyperkalemia risk associated with antihypertensive combination regimens including RASi-based dual antihypertensive treatment in CKD patients are warranted to improve patient prognosis.

The risk of ESRD diagnosis was statistically insignificant among antihypertensive treatment regimens. As CKD being a progressive chronic disorder, disease progression to ESRD may inevitable in many patients with the estimated probability of 52% in 10 years^36^. However, the time to ESRD diagnosis may be different as numerous patient- and disease-specific factors play crucial roles in ESRD progressions, and the studies suggest that those with younger age, female sex, diabetes, or dyslipidemia are more likely to have accelerated progression to ESRD^37,38^. Considering the substantial clinical adverse outcomes in ESRD patients, delaying ESRD progression itself may be acknowledged as valuable clinical benefits in CKD patients^39^. Thus, further studies investigating the impact of various antihypertensive treatment regimens on the time to ESRD diagnosis are warranted to ensure favorable prognoses in CKD patients.

Diabetes is also a major etiologic comorbidity for CKD diagnosis and progression, and BP management in CKD patients with diabetes is also pivotal as more than 80% of these patients are diagnosed with hypertension^40^. The guidelines recommend RASi-based antihypertensive treatment regimens regardless of diabetes status^1,10^. However, despite the significant influence of diabetes on accelerated disease progression in CKD patients, the studies investigating clinical outcomes of diverse antihypertensive treatment regimens are still limited^38^. Among 16 clinical trials included in this study, 9 clinical trials evaluated outcomes from 7 different antihypertensive regimen in CKD patients with diabetes: ACEI monotherapy, ARB monotherapy, ACEI-based spironolactone, ARB-based spironolactone, ARB and ACEI combination, and ARB-based renin inhibitor. The risk of major CVD events was substantially higher in ACEI + SPR (OR 5.53; 95% CI 1.05–29.19) when compared to ARB monotherapy (reference), the most prescribed antihypertensive regimen in Korea (Supplementary Figure S2)^9,41^. Meanwhile, the risk of hyperkalemia was similar among the treatment regimens when referenced with ARB monotherapy (Supplementary Figure S3). Nonetheless, considering that administration of RASi with spironolactone is rarely recommended in CKD patients, the only viable antihypertensive treatment regimens recommended by the current guidelines are ACEI monotherapy and ARB monotherapy in this analysis, implying the limited number of clinical trials in CKD patients with diabetes. Hence, further studies investigating clinical outcomes such as BP control and major CVD incidences associated different RASi-based antihypertensive treatment regimens in CKD patients with diabetes are required.

To the best of our knowledge, this is the first network meta-analysis investigating clinical outcomes of RASi-based dual antihypertensive regimens in non-dialysis CKD patients. We comprehensively compared cardiovascular outcomes including major CVD risks and BP controls as well as other clinical outcomes such as the risk of hyperkalemia, ESRD and all-cause mortality and demonstrated substantial clinical benefits from ARB-based CCB combination in non-dialysis CKD patients. However, considering more than 45% of advanced CKD patients discontinue RASi therapy within 1-year secondary to CKD progression, hospitalization for acute kidney injury, hyperkalemia, and the presence of multiple comorbidities, noticeably large number of CKD patients require antihypertensive agents other than RASi^31,42^. Thus, we additionally included 3 non-RASi-based antihypertensive regimens, CCB monotherapy, combination of CCB and BB, and combination of CCB and TZD, in the analysis based on prespecified PICOST-SD and demonstrated favorable effects of these regimens on major CVD risks. Nonetheless, the apparent benefits of non-RASi-based dual antihypertensive treatment should be thoroughly evaluated due to extremely limited evidences.

This study has several limitations. The included studies had differences in study designs, outcome measurements, and study follow-up durations, consequently increasing heterogeneity across the studies. Moreover, due to the vulnerability associated with CKD, small number of clinical trials as well as study participants may also play as limitation. However, considering that the majority of CKD patients are not eligible for the clinical trials, our study might contribute to the current body of literature on selection guidance on optimal hypertension pharmacotherapy in non-dialysis CKD patients to properly manage BP, thereby ameliorating the risk of major CVD events. Additionally, we included antihypertensive treatment regimens that are not currently recommended by the guidelines, combination of ACEI and ARB for example, to perform stepwise comparisons for network meta-analysis because more than half of the included studies had combination of ARB and ACEI regimen as study arm. Although exclusion of patients on impractical regimens may decrease the number of participants, this study manifested that combination of ARB and ACEI treatment attenuates the risk of major CVD despite comparable hyperkalemia risk with other antihypertensive regimens in non-dialysis CKD patients.

## Conclusion

RASi-based dual antihypertensive regimen with ARB and CCB provided the most significant BP reduction as well as the lowest odds of major CVD events than other RASi-based antihypertensive regimens. However, the odds of hyperkalemia, ESRD progression, and all-cause mortality were statistically insignificant among various RASi-based antihypertensive regimens in CKD patients.

## Methods

### Data sources and search strategy

This study was conducted according to the Preferred Reporting Items for Systematic Reviews and Meta-Analysis (PRISMA) 2020 and PRISMA-Network Meta-Analyses (PRISMA-NMA) guidelines (Supplementary Table S1 and S2^43,44^. Cochrane Library, MEDLINE (PubMed), and Scopus were searched from the inception to March 7 2023. The prespecified Medical Subject Headings (MeSH) and keywords were utilized for the initial database search: ‘angiotensin-converting enzyme inhibitors’, ‘angiotensin receptor antagonists’, ‘chronic kidney disease’, ‘hypertension’, and ‘renal insufficiency’. We also used ‘combin*’ as keyword to identify studies evaluating efficacy and safety of antihypertensive combination therapy containing either ACEI or ARB. The full database search terms and strategies are listed in Supplementary Table S3. The prespecified search filters were ‘clinical trials’, ‘humans’ and ‘English’. The manual search of the references from the eligible articles, otherwise referred to as snowball search, was performed to identify additional studies that meet eligible criteria. The protocol of this systematic review and network meta-analysis were registered in the International Prospective Register of Systematic Reviews (PROSPERO No. CRD42022365927).

### Study selection and data extraction

The PICOST-SD (patient, intervention, control, outcomes, setting, time, and study design) is prespecified as follows: patients with HTN and non-dialysis CKD (stage 3 to 5), intervention with RASi-based (either ACEI or ARB) dual antihypertensive treatment, both inpatient and outpatients with treatment duration ≥ 24 weeks, and head-to-head RCT. Control was prespecified as any hypertensive treatment regimens including monotherapy and dual antihypertensive therapy that were directly compared with the intervention in the head-to-head RCT. Two reviewers independently screened titles/abstracts for eligibility of all identified studies from the initial database. The prespecified eligibility criteria of study inclusion included (1) head-to-head randomized controlled trials (RCT), (2) studies evaluating efficacy and safety of dual antihypertensive treatment regimens containing either ACEIs or ARBs as intervention in non-dialysis patients with CKD stage 3 to 5, (3) antihypertensive medications prescribed for ≥ 24 weeks, and (4) studies published in English. Duplicated studies, placebo-controlled RCTs, case reports, cross-over designed studies, observational studies, preclinical studies (in-vivo or in-vitro), abstracts including conference abstracts, study protocols, proceedings, reviews, and studies without available full-texts were excluded. Any disagreements on study eligibility were discussed until a consensus was reached. Cohen’s kappa statistic was calculated to determine interrater reliability for study selection and revealed kappa value of 0.88, implying strong level of agreement^45^.

The primary outcome of interest was major CVD events identified as MI, stroke, cardiovascular (CV) death, and CV-related hospitalization due to heart failure or angina as these have been identified as the most significant cause of mortality in CKD patient^6^. Moreover, other clinical outcomes including reduction in systolic blood pressure (SBP) and diastolic blood pressure (DBP), hyperkalemia, newly diagnosed ESRD (ESRD progression), and all-cause mortality, otherwise referred to as death of any cause were also investigated. Following information was extracted from the eligible study: study characteristics (author and year of publication), study population (number of patients and comorbidities), study intervention and comparators, and treatment duration. We extracted the number of patients experienced the outcomes of the study for dichotomous outcomes. For continuous outcomes such as SBP and DBP, we extracted the mean and standard deviation (SD) of each intervention.

### Study quality assessments

Two reviewers assessed the quality of included studies, and any disagreements on study eligibility were resolved by the third reviewer (SJR). Risk of bias of included studies was assessed in accordance with Cochrane Risk of Bias version 2.0 (RoB 2) tool^46^, and each study were scored as low, some concerns (unclear), or high risk in the following domains: randomization process, deviations from the intended interventions, missing outcome data, measurements of the outcomes, and selection of the reported results. Any disagreements on the quality assessments were discussed until a consensus was reached. Studies were classified as low risk of bias if three or more domains were identified as low risk, whereas studies with at least two domains with high risk were classified as high-risk studies. The quality of evidences was assessed based on Cochrane GRADE guideline for each outcome^47^.

### Statistical analysis and data synthesis

Pooled traditional pair-wise analyses on the outcomes were performed using ‘netmeta’ package in R (version 4.1.0.). Frequentist network meta-analyses were conducted to simultaneously compare the outcomes of interest by integrating direct and indirect effects of each RASi-based dual antihypertensive treatment regimen against a control group generated by the network meta-analysis in CKD patients^48^. ARB-based dual antihypertensive regimen with calcium channel blocker (CCB) was applied as the control antihypertensive treatment regimen for network analysis^9,41^. Dichotomous variables including major CVD events, ESRD diagnosis, all-cause mortality, and hyperkalemia were analyzed with a Mantel–Haenszel random effects model and odds ratio (OR) with 95% confidence intervals (CI) were calculated for each antihypertensive regimen. Changes in SBP and DBP were evaluated as weighted standard mean differences (SMD) with 95% CI. The heterogeneity and inconsistency of included studies were assessed with I^2^ index and Cochran’s Q, respectively. I^2^ index > 50% was considered as high heterogeneity and any *P* value < 0.05 was considered as significant inconsistency^49^. Egger’s test was performed to detect any publication of bias, and any *P* value > 0.05 implies a low risk of publication bias. The “netrank” function was utilized to assess the ranking of relative efficacy and safety of antihypertensive regimens in CKD patients, and the calculated *P* value was equivalent to the Surface Under the Cumulative Ranking (SCURA): the higher the *P* value, the better the rank of the antihypertensive treatment regimen^50^. The “netleague” function was utilized to create league tables for each outcome. All *P* values were calculated by two-sided tests, and statistical significance was determined by *P* values < 0.05.

## Supplementary Information

Supplementary Information 1.

## Data Availability

All data generated or analysed during this study are included in this published article [and its supplementary information files].
