# Supplementary material for: Clinical outcomes of renin angiotensin system inhibitor-based dual antihypertensive regimens in chronic kidney disease: a network meta-analysis
Source: Sci Rep. 2023 Apr 7;13:5727. doi: 10.1038/s41598-023-32266-4 (PMC10082011; doi:10.1038/s41598-023-32266-4)
Supplement: Supplementary file 1 — Supplementary Information 1. [file 41598_2023_32266_MOESM1_ESM.pdf]

PRISMA 2020 Checklist

Table S1. PRISMA 2020 checklist

Section and Topic	Item #	Checklist item	Location where item is reported
**TITLE**			
Title	1	Identify the report as a systematic review.	Page 1
**ABSTRACT**			
Abstract	2	See the PRISMA 2020 for Abstracts checklist.	Page 1
**INTRODUCTION**			
Rationale	3	Describe the rationale for the review in the context of existing knowledge.	Page 1
Objectives	4	Provide an explicit statement of the objective(s) or question(s) the review addresses.	Page 1-2
**METHODS**			
Eligibility criteria	5	Specify the inclusion and exclusion criteria for the review and how studies were grouped for the syntheses.	Page 10
Information sources	6	Specify all databases, registers, websites, organisations, reference lists and other sources searched or consulted to identify studies. Specify the date when each source was last searched or consulted.	Page 10
Search strategy	7	Present the full search strategies for all databases, registers and websites, including any filters and limits used.	Page 10<br>Supplementary Table S3
Selection process	8	Specify the methods used to decide whether a study met the inclusion criteria of the review, including how many reviewers screened each record and each report retrieved, whether they worked independently, and if applicable, details of automation tools used in the process.	Page 10
Data collection process	9	Specify the methods used to collect data from reports, including how many reviewers collected data from each report, whether they worked independently, any processes for obtaining or confirming data from study investigators, and if applicable, details of automation tools used in the process.	Page 10
Data items	10a	List and define all outcomes for which data were sought. Specify whether all results that were compatible with each outcome domain in each study were sought (e.g. for all measures, time points, analyses), and if not, the methods used to decide which results to collect.	Page 10
	10b	List and define all other variables for which data were sought (e.g. participant and intervention characteristics, funding sources). Describe any assumptions made about any missing or unclear information.	Page 10
Study risk of bias assessment	11	Specify the methods used to assess risk of bias in the included studies, including details of the tool(s) used, how many reviewers assessed each study and whether they worked independently, and if applicable, details of automation tools used in the process.	Page 10
Effect measures	12	Specify for each outcome the effect measure(s) (e.g. risk ratio, mean difference) used in the synthesis or presentation of results.	Page 10-11
Synthesis methods	13a	Describe the processes used to decide which studies were eligible for each synthesis (e.g. tabulating the study intervention characteristics and comparing against the planned groups for each synthesis (item #5)).	Page 10-11
	13b	Describe any methods required to prepare the data for presentation or synthesis, such as handling of missing summary statistics, or data conversions.	Page 10-11

PRISMA 2020 Checklist

Section and Topic	Item #	Checklist item	Location where item is reported
	13c	Describe any methods used to tabulate or visually display results of individual studies and syntheses.	Page 10-11
	13d	Describe any methods used to synthesize results and provide a rationale for the choice(s). If meta-analysis was performed, describe the model(s), method(s) to identify the presence and extent of statistical heterogeneity, and software package(s) used.	Page 10-11
	13e	Describe any methods used to explore possible causes of heterogeneity among study results (e.g. subgroup analysis, meta-regression).	Page 10-11
	13f	Describe any sensitivity analyses conducted to assess robustness of the synthesized results.	Page 10-11
Reporting bias assessment	14	Describe any methods used to assess risk of bias due to missing results in a synthesis (arising from reporting biases).	Page 10
Certainty assessment	15	Describe any methods used to assess certainty (or confidence) in the body of evidence for an outcome.	Page 10
**RESULTS**			
Study selection	16a	Describe the results of the search and selection process, from the number of records identified in the search to the number of studies included in the review, ideally using a flow diagram.	Page 2
	16b	Cite studies that might appear to meet the inclusion criteria, but which were excluded, and explain why they were excluded.	Page 2, Table 1
Study characteristics	17	Cite each included study and present its characteristics.	Page 2, Table 1
Risk of bias in studies	18	Present assessments of risk of bias for each included study.	Page 2<br>Figure 3<br>Supplementary Figure S1
Results of individual studies	19	For all outcomes, present, for each study: (a) summary statistics for each group (where appropriate) and (b) an effect estimate and its precision (e.g. confidence/credible interval), ideally using structured tables or plots.	Page 2-3
Results of syntheses	20a	For each synthesis, briefly summarise the characteristics and risk of bias among contributing studies.	Page 2-8
	20b	Present results of all statistical syntheses conducted. If meta-analysis was done, present for each the summary estimate and its precision (e.g. confidence/credible interval) and measures of statistical heterogeneity. If comparing groups, describe the direction of the effect.	Page 2-8
	20c	Present results of all investigations of possible causes of heterogeneity among study results.	Page 2-8
	20d	Present results of all sensitivity analyses conducted to assess the robustness of the synthesized results.	Page 2-8
Reporting biases	21	Present assessments of risk of bias due to missing results (arising from reporting biases) for each synthesis assessed.	Page 2-8<br>Supplementary Figure S1
Certainty of	22	Present assessments of certainty (or confidence) in the body of evidence for each outcome assessed.	Page 2-8

PRISMA 2020 Checklist

Section and Topic	Item #	Checklist item	Location where item is reported
evidence			
**DISCUSSION**			
Discussion	23a	Provide a general interpretation of the results in the context of other evidence.	Page 3-8
	23b	Discuss any limitations of the evidence included in the review.	Page 3-8
	23c	Discuss any limitations of the review processes used.	Page 3-8
	23d	Discuss implications of the results for practice, policy, and future research.	Page 3-8
**OTHER INFORMATION**			
Registration and protocol	24a	Provide registration information for the review, including register name and registration number, or state that the review was not registered.	Page 10
	24b	Indicate where the review protocol can be accessed, or state that a protocol was not prepared.	PROSPERO (page 10)
	24c	Describe and explain any amendments to information provided at registration or in the protocol.	Not appliable
Support	25	Describe sources of financial or non-financial support for the review, and the role of the funders or sponsors in the review.	Page 12
Competing interests	26	Declare any competing interests of review authors.	Page 12
Availability of data, code and other materials	27	Report which of the following are publicly available and where they can be found: template data collection forms; data extracted from included studies; data used for all analyses; analytic code; any other materials used in the review.	Page 12

From: Page MJ, McKenzie JE, Bossuyt PM, Boutron I, Hoffmann TC, Mulrow CD, et al. The PRISMA 2020 statement: an updated guideline for reporting systematic reviews. BMJ 2021;372:n71. doi: 10.1136/bmj.n71

For more information, visit: http://www.prisma-statement.org/

Table S2. PRISMA NMA checklist

PRISMA NMA Checklist of Items to Include When Reporting A Systematic Review Involving a Network Meta-analysis

Section/Topic	Item #	Checklist Item	Reported on Page #
**TITLE**			
Title	1	Identify the report as a systematic review *incorporating a network meta-analysis (or related form of meta-analysis).*	***Page 1***
**ABSTRACT**			
Structured summary	2	Provide a structured summary including, as applicable:<br>**Background:** main objectives<br>**Methods:** data sources; study eligibility criteria, participants, and interventions; study appraisal; and *synthesis methods, such as network meta-analysis.*<br>**Results:** number of studies and participants identified; summary estimates with corresponding confidence/credible intervals; *treatment rankings may also be discussed. Authors may choose to summarize pairwise comparisons against a chosen treatment included in their analyses for brevity.*<br>**Discussion/Conclusions:** limitations; conclusions and implications of findings.<br>**Other:** primary source of funding; systematic review registration number with registry name.	*Page 1*
**INTRODUCTION**			
Rationale	3	Describe the rationale for the review in the context of what is already known*, including mention of why a network meta-analysis has been conducted.*_	***Page 2***
Objectives	4	Provide an explicit statement of questions being addressed, with reference to participants, interventions, comparisons, outcomes, and study design (PICOS).	Page 2-3
**METHODS**			
Protocol and registration	5	Indicate whether a review protocol exists and if and where it can be accessed (e.g., Web address); and, if available, provide registration information, including registration number.	Page 10
Eligibility criteria	6	Specify study characteristics (e.g., PICOS, length of follow-up) and report characteristics (e.g., years considered, language, publication status) used as criteria for eligibility, giving rationale. *Clearly describe eligible treatments included in the*	***Page 10***

		*treatment network, and note whether any have been clustered or merged into the same node (with justification).*_	
Information sources	7	Describe all information sources (e.g., databases with dates of coverage, contact with study authors to identify additional studies) in the search and date last searched.	Page 10
Search	8	Present full electronic search strategy for at least one database, including any limits used, such that it could be repeated.	Page 10 (Supplementary Table S3)
Study selection	9	State the process for selecting studies (i.e., screening, eligibility, included in systematic review, and, if applicable, included in the meta-analysis).	Page 10
Data collection process	10	Describe method of data extraction from reports (e.g., piloted forms, independently, in duplicate) and any processes for obtaining and confirming data from investigators.	Page 10
Data items	11	List and define all variables for which data were sought (e.g., PICOS, funding sources) and any assumptions and simplifications made.	Page 10
**Geometry of the network**	**S1**	Describe methods used to explore the geometry of the treatment network under study and potential biases related to it. This should include how the evidence base has been graphically summarized for presentation, and what characteristics were compiled and used to describe the evidence base to readers.	***Page 10-11 Figure 4***
Risk of bias within individual studies	12	Describe methods used for assessing risk of bias of individual studies (including specification of whether this was done at the study or outcome level), and how this information is to be used in any data synthesis.	Page 10
Summary measures	13	State the principal summary measures (e.g., risk ratio, difference in means). *Also describe the use of additional summary measures assessed, such as treatment rankings and surface under the cumulative ranking curve (SUCRA) values, as well as modified approaches used to present summary findings from meta-analyses.*	Page 10-11
Planned methods of analysis	14	Describe the methods of handling data and combining results of studies for each network meta-analysis. This should include, but not be limited to:<br>• *Handling of multi-arm trials;*<br>• *Selection of variance structure;*<br>• *Selection of prior distributions in Bayesian analyses; and*<br>• *Assessment of model fit.*	Page 10 -11
**Assessment of Inconsistency**	**S2**	Describe the statistical methods used to evaluate the agreement of direct and indirect evidence in the treatment network(s) studied. Describe efforts taken to address its presence when found.	Page 10-11
Risk of bias across studies	15	Specify any assessment of risk of bias that may affect the cumulative evidence (e.g., publication	**Page 10-11**

		bias, selective reporting within studies).	
Additional analyses	16	Describe methods of additional analyses if done, indicating which were pre-specified. This may include, but not be limited to, the following: • Sensitivity or subgroup analyses; • Meta-regression analyses; • *Alternative formulations of the treatment network; and* • *Use of alternative prior distributions for Bayesian analyses (if applicable).*_	***Page 10***
**RESULTS†**			
Study selection	17	Give numbers of studies screened, assessed for eligibility, and included in the review, with reasons for exclusions at each stage, ideally with a flow diagram.	Page 2
**Presentation of network structure**	**S3**	Provide a network graph of the included studies to enable visualization of the geometry of the treatment network.	***Figure 2*** ***Page 4***
**Summary of network geometry**	**S4**	Provide a brief overview of characteristics of the treatment network. This may include commentary on the abundance of trials and randomized patients for the different interventions and pairwise comparisons in the network, gaps of evidence in the treatment network, and potential biases reflected by the network structure.	***Page 2-4*** ***Figure 2***
Study characteristics	18	For each study, present characteristics for which data were extracted (e.g., study size, PICOS, follow-up period) and provide the citations.	Page 2
Risk of bias within studies	19	Present data on risk of bias of each study and, if available, any outcome level assessment.	Page 2-4 Figure 3 Supplementary Figure S1
Results of individual studies	20	For all outcomes considered (benefits or harms), present, for each study: 1) simple summary data for each intervention group, and 2) effect estimates and confidence intervals. *Modified approaches may be needed to deal with information from larger networks.*	Page 2-8
Synthesis of results	21	Present results of each meta-analysis done, including confidence/credible intervals. *In larger networks, authors may focus on comparisons versus a particular comparator (e.g. placebo or standard care), with full findings presented in an appendix. League tables and forest plots may be considered to summarize pairwise comparisons.* If additional summary measures were explored (such as	***Page 2-8*** ***Figure 4-5***

		treatment rankings), these should also be presented.	
**Exploration for inconsistency**	**S5**	Describe results from investigations of inconsistency. This may include such information as measures of model fit to compare consistency and inconsistency models, *P* values from statistical tests, or summary of inconsistency estimates from different parts of the treatment network.	***Page 2-8***
Risk of bias across studies	22	Present results of any assessment of risk of bias across studies for the evidence base being studied.	Page 2
Results of additional analyses	23	Give results of additional analyses, if done (e.g., sensitivity or subgroup analyses, meta-regression analyses*, alternative network geometries studied, alternative choice of prior distributions for Bayesian analyses,* and so forth).	***Page 2-8***
**DISCUSSION**			
Summary of evidence	24	Summarize the main findings, including the strength of evidence for each main outcome; consider their relevance to key groups (e.g., healthcare providers, users, and policy-makers).	Page 4-10
Limitations	25	Discuss limitations at study and outcome level (e.g., risk of bias), and at review level (e.g., incomplete retrieval of identified research, reporting bias). *Comment on the validity of the assumptions, such as transitivity and consistency. Comment on any concerns regarding network geometry (e.g., avoidance of certain comparisons).*	Page 9-10
Conclusions	26	Provide a general interpretation of the results in the context of other evidence, and implications for future research.	Page 10
**FUNDING**			
Funding	27	Describe sources of funding for the systematic review and other support (e.g., supply of data); role of funders for the systematic review. This should also include information regarding whether funding has been received from manufacturers of treatments in the network and/or whether some of the authors are content experts with professional conflicts of interest that could affect use of treatments in the network.	***Page 12***

PICOS = population, intervention, comparators, outcomes, study design.
* Text in italics indicateS wording specific to reporting of network meta-analyses that has been added to guidance from the PRISMA statement.
† Authors may wish to plan for use of appendices to present all relevant information in full detail for items in this section.

Table S3. Database search terms

<table>
<tr><th>Database</th><th>Search terms and results</th></tr>
<tr><td>1) Pubmed</td><td>((chronic kidney disease[MeSH Terms]) AND (hypertension[MeSH Terms]) AND ((angiotensin converting enzyme inhibitors[MeSH Terms])) OR (angiotensin receptor blockers[MeSH Terms])) AND (combin*)

(((renal insufficiency[MeSH Terms]) AND (hypertension[MeSH Terms]) AND ((angiotensin converting enzyme inhibitors[MeSH Terms])) OR (angiotensin receptor blockers[MeSH Terms]))) AND (combin*)</td></tr>
<tr><td>2) Cochrane Library</td><td><table>
<tr><td>#1</td><td>MeSH descriptor: [Renal Insufficiency] explode all trees</td></tr>
<tr><td>#2</td><td>MeSH descriptor: [Renal Insufficiency, Chronic] explode all trees</td></tr>
<tr><td>#3</td><td>MeSH descriptor: [Hypertension] explode all trees</td></tr>
<tr><td>#4</td><td>MeSH descriptor: [Angiotensin-Converting Enzyme Inhibitors] explode all trees</td></tr>
<tr><td>#5</td><td>MeSH descriptor:[Angiotensin Receptor Antagonists] explode all trees</td></tr>
<tr><td>#6</td><td>Combin* (Limits: in Trials)</td></tr>
<tr><td>#7</td><td>((#1 and #2) and #3 and (#4 or #5) and #6)</td></tr>
<tr><td>#8</td><td>Chronic kidney disease (Limits: in Trials)</td></tr>
<tr><td>#9</td><td>Hypertension (Limits: in Trials)</td></tr>
<tr><td>#10</td><td>((#1 or #2 or #8) and (#3 or #9) and (#4 or #5) and #6)</td></tr>
</table></td></tr>
<tr><td>3) Scopus</td><td>((ALL ("renal insufficien*") OR ALL ("chronic kidney disease") OR ALL ("chronic renal disease") OR ALL ("CKD") AND ALL ("Hypertension"))) AND ((ALL ("angiotensin converting enzyme inhibitor*") OR ALL ("angiotensin receptor blocker*") OR ALL ("angiotensin receptor antagonist") OR ALL ("ACEI") OR ALL ("ARB"))) AND (TITLE-ABS-KEY ("combin*"))

((ALL ("renal insufficien*") OR ALL ("chronic kidney disease") OR ALL ("chronic renal disease") OR ALL ("CKD") AND ALL ("Hypertension"))) AND ((ALL ("angiotensin converting enzyme inhibitor*") OR ALL ("angiotensin receptor blocker*") OR ALL ("angiotensin receptor antagonist") OR ALL ("ACEI") OR ALL ("ARB"))) AND (TITLE-ABS-KEY ("combin*")) AND (LIMIT-TO (EXACTKEYWORD , "Major Clinical Study"))
(Limits: Keyword “Major Clinical Study”)</td></tr>
</table>

Figure S1. Study quality assessment

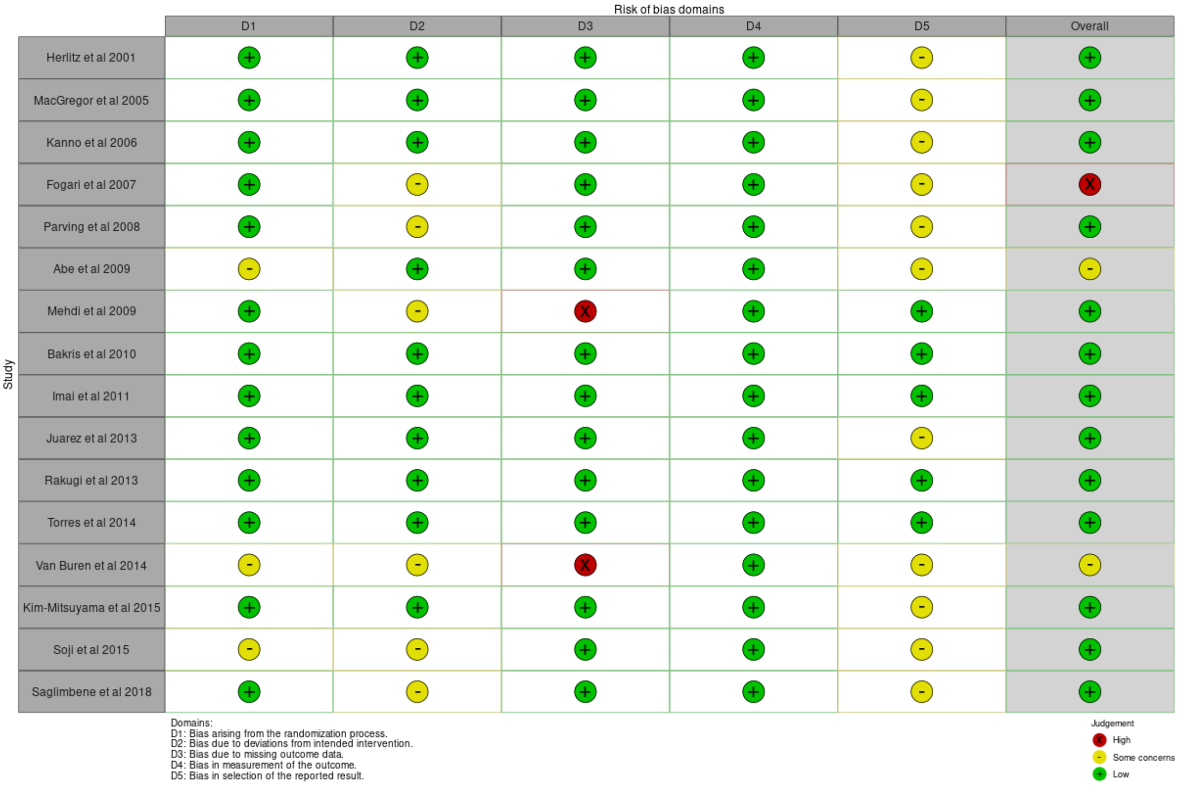

Figure S2. Forest plots of antihypertensive treatment regimens on major CVD in CKD patients with DM. A) major CVD events and B) hyperkalemia

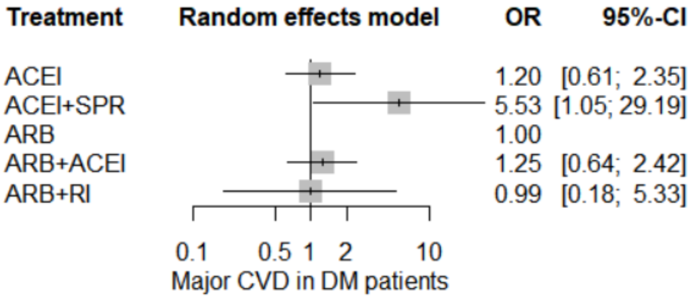

ACEI	0.13 [0.01; 1.28]	1.04 [0.50; 2.15]	0.96 [0.59; 1.56]	
0.22 [0.04; 1.05]	ACEI+SPR		3.43 [0.58; 20.22]	
1.20 [0.61; 2.35]	**5.53 [1.05; 29.19]**	ARB	0.71 [0.35; 1.43]	1.01 [0.19; 5.44]
0.96 [0.59; 1.56]	4.42 [0.94; 20.88]	0.80 [0.41; 1.55]	ARB+ACEI	
1.21 [0.20; 7.40]	5.58 [0.52; 59.52]	1.01 [0.19; 5.44]	1.26 [0.21; 7.70]	ARB+RI

Figure S3. Forest plot of antihypertensive treatment regimens on hyperkalemia in CKD patients with DM

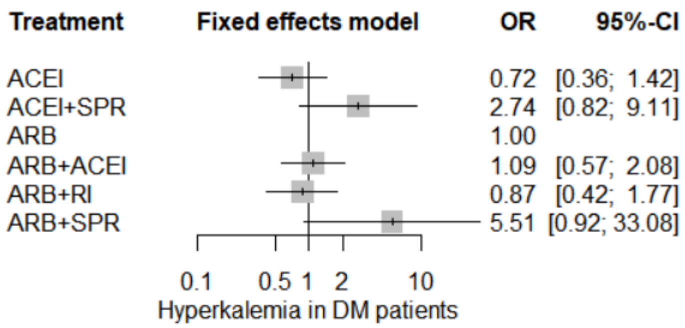

ACEI	**0.07 [0.01; 0.41]**	0.89 [0.38; 2.06]	0.64 [0.37; 1.10]		
**0.24 [0.07; 0.82]**	ACEI+SPR		1.72 [0.51; 5.86]		
0.72 [0.33; 1.56]	2.93 [0.76; 11.24]	ARB	1.03 [0.47, 2.25]	1.15 [0.47; 2.84]	0.18 [0.03; 1.15]
0.64 [0.37; 1.10]	2.61 [0.83; 8.23]	0.89 [0.42; 1.88]	ARB+ACEI		
0.83 [0.25; 2.73]	3.38 [0.67; 17.06]	1.15 [0.47; 2.84]	1.29 [0.40;4.16]	ARB+RI	
0.13 [0.02; 0.96]	0.53 [0.05; 5.22]	0.18 [0.03; 1.15]	0.20 [0.03; 1.49]	0.16 [0.02; 1.23]	ARB+SPR
